# Waste reduction decreases rat activity from peri-urban environment

**DOI:** 10.1371/journal.pone.0308917

**Published:** 2024-11-13

**Authors:** Ishana Shukla, Christopher C. Wilmers

**Affiliations:** 1 Department of Ecology and Evolutionary Biology, University of California, Santa Cruz, Santa Cruz, California, United States of America; 2 Department of Environmental Studies, University of California, Santa Cruz, Santa Cruz, California, United States of America; MARE – Marine and Environmental Sciences Centre, PORTUGAL

## Abstract

Globally, species in the genus *Rattus* (specifically *Rattus rattus and Rattus norvegicus)*, are some of the most influential invasive taxa due to their high rates of competitive exclusion and large dietary breadth. However, the specific foraging strategies of urban-adjacent populations remain largely unknown. We examined *Rattus* spp. dependency on human food supplementation in a population on adjacent non-developed (or peri-urban) land. Via linear regression modeling, we measured rodent activity changes between native and invasive species before and after a decrease in human supplementation due to the COVID-19 lockdown in Santa Cruz, California, USA. We documented invasive rat activity via camera traps in normal (pre-COVID lockdown) conditions near dining halls and similar waste sources, and again under COVID lockdown conditions when sources of human supplementation were drastically decreased. After 120 trap nights we found a significant decrease (p < 0.001) in *Rattus* activity after the removal of human refuse, while native small mammal activity remained unchanged (p = 0.1). These results have strong conservation implications, as they support the hypothesis that proper waste management is an effective, less-invasive form of population control over conventional rodenticides.

## Introduction

Human refuse attracts synanthropic rodent species, and these local populations can use this supplementation to bolster their shelter opportunities, reduce predation risk, and notably, supplement their diet [1]. This supplementation can promote population growth of invasive species and/or lead to high equilibrium levels of such species [2, 3]. While not all species in a community will benefit from human supplementation, the indirect and direct effects of these benefits can expand outside the species level and impact large portions of the food web [4].

Two species in the genus *Rattus* (i.e., the black rat *Rattus rattus* and the brown rat *Rattus norvegicus)* currently stand as some of the most widespread invasive taxa worldwide, and their presence is documented on all continents, save for Antarctica [5, 6]. Known for their resourceful foraging strategies, these invasive *Rattus* species can affect native populations of small rodents through both predation and competitive exclusion [7, 8]. Generally, invasive rats are classified as facultative foragers due to their varied diet. Despite their generalist reputation, invasive rat dietary breadth between island and mainland populations has the potential to differ significantly due to differences in resource types [9]. Island populations of invasive rats show extreme resourcefulness and a large dietary breadth, but the degree to which mainland peri-urban populations’ foraging strategies differ remains largely unexplored [7, 10].

Prior studies suggest that strictly urban populations of invasive rats have a dietary preference for human refuse–a pattern that spans multiple geographies [11, 12]. Current urban invasive rat literature centers largely on pest control management and reiterates strong ties between human refuse and invasive rat activity [10, 13, 14]. While access to food, water, and shelter all contribute to a rat’s urban habitat selection, food supplementation is thought to be paramount in determining population levels [15]. Parallel urban pandemic studies that monitored rat-bait stations or pest control complaints in cities report overall mixed invasive rat activity, but most suggest a redistribution of population structure [16–18]. Still, these studies focus on completely urbanized populations, and lesser known is the degree to which peri-urban populations depend on human supplementation [19]. In ideal circumstances, foraging dependency would be tested with removal experiments, but these experiments are generally difficult to carry out. The experimental site would have to undergo extreme environmental manipulation and be monitored for multiple seasons to account for any relaxation time for a population to reach a new equilibrium. Nonetheless, these experiments can provide results on obligate foraging and specialized dependency in a population [20]. Here, with a natural removal experiment, we test the hypothesis that established, peri-urban populations of invasive *Rattus* species are human-obligate foragers and are dependent on nutritional supplementation from human refuse to maintain population levels. We predict little to no changes in native small mammal activity, as we hypothesize that native small mammals are less reliant on this human supplementation. We test this by taking advantage of an abrupt reduction in human food supplementation via COVID-19 (hereafter COVID) lockdowns in Santa Cruz County, California, USA, and analyzed the change in invasive rat activity via camera trapping.

## Materials and methods

### Study area and removal experiment

**Fig 1:**
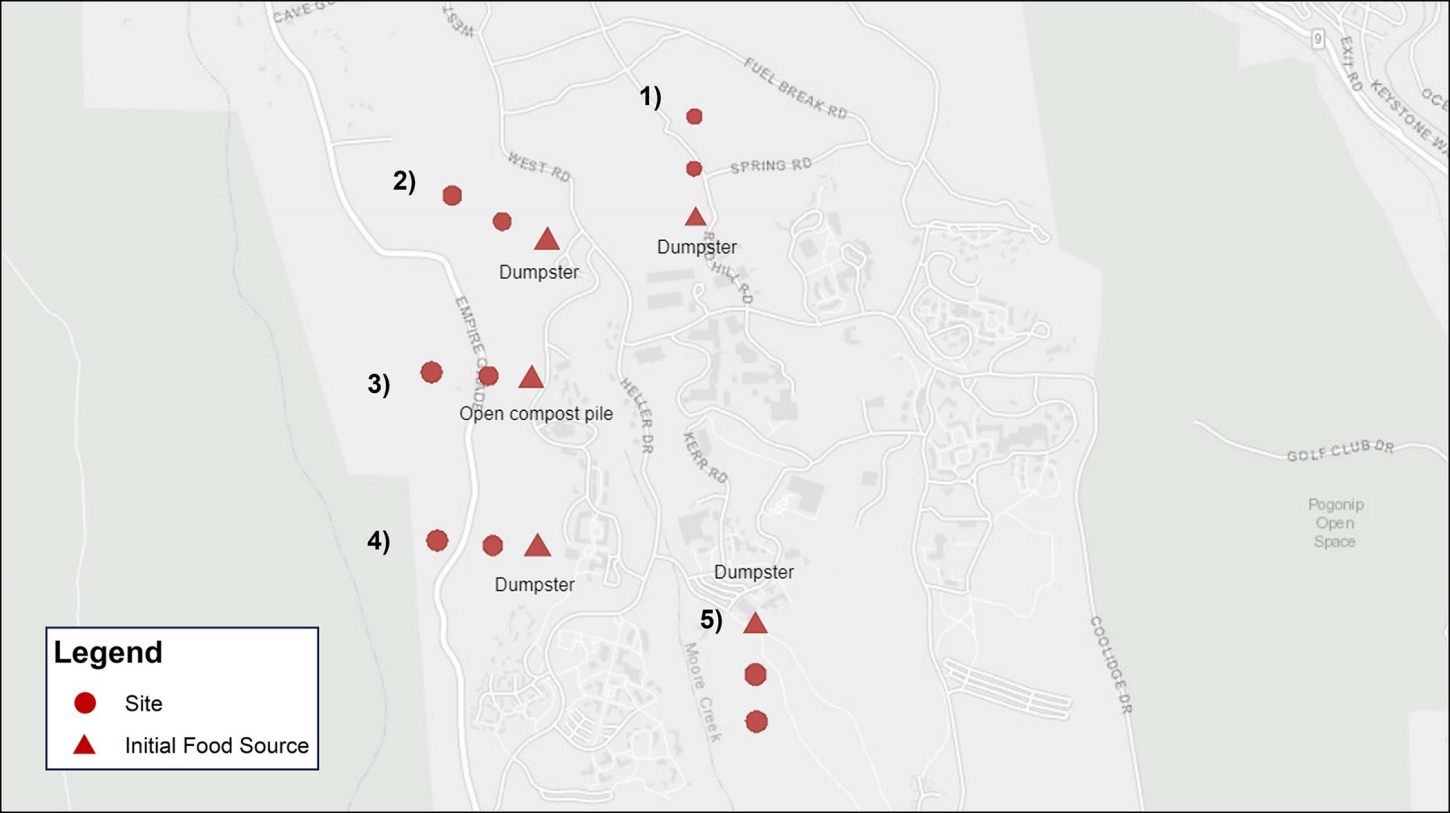
A 1:2000 scale map of the 2.12 km^2^ study area: The UC Santa Cruz campus. Sites remained constant across Trapping Periods 1 and 2. Cameras placed at initial food sources (labeled) are marked with a triangle, while cameras placed at each sequential site (75 m and 150 m toward natural habitat, respectively) are marked with a circle. Site 1 was located in pure redwood habitat, Sites 2 and 3 were located in mixed redwood habitats, and Sites 4 and 5 were located in mixed oak habitats.

All data were collected on the University of California, Santa Cruz (UC Santa Cruz) campus and adjacent non-developed land (hereafter “peri-urban”), spanning a total area of 2.12 km^2^ (Fig 1). A number of native small rodents are found throughout the UC Santa Cruz campus, including deer mice *Peromyscus californicus*, pinyon mice *Peromyscus trueii*, and dusky-footed woodrats *Neotoma fuscipes* [21]. The peri-urban land on campus is located on a natural reserve and primarily consists of three forest types: mixed oak woodland, redwood, and mixed redwood habitat. The mixed oak woodland is primarily composed of tanoak *Notholithocarpus densiflorus*, pacific madrone *Arbutus menziesii* and Shreve oak *Quercus parvula var*. *shrevei*. The mixed redwood is primarily composed of coastal redwood *Sequoia sempervirens*, and douglas fir *Pseudotsuga menziesii*. The redwood habitat is almost entirely composed of coastal redwood. Adjoining these forests is meadow habitat interspersed with coyote brush *Baccharis pilularis*.

**Fig 2:**
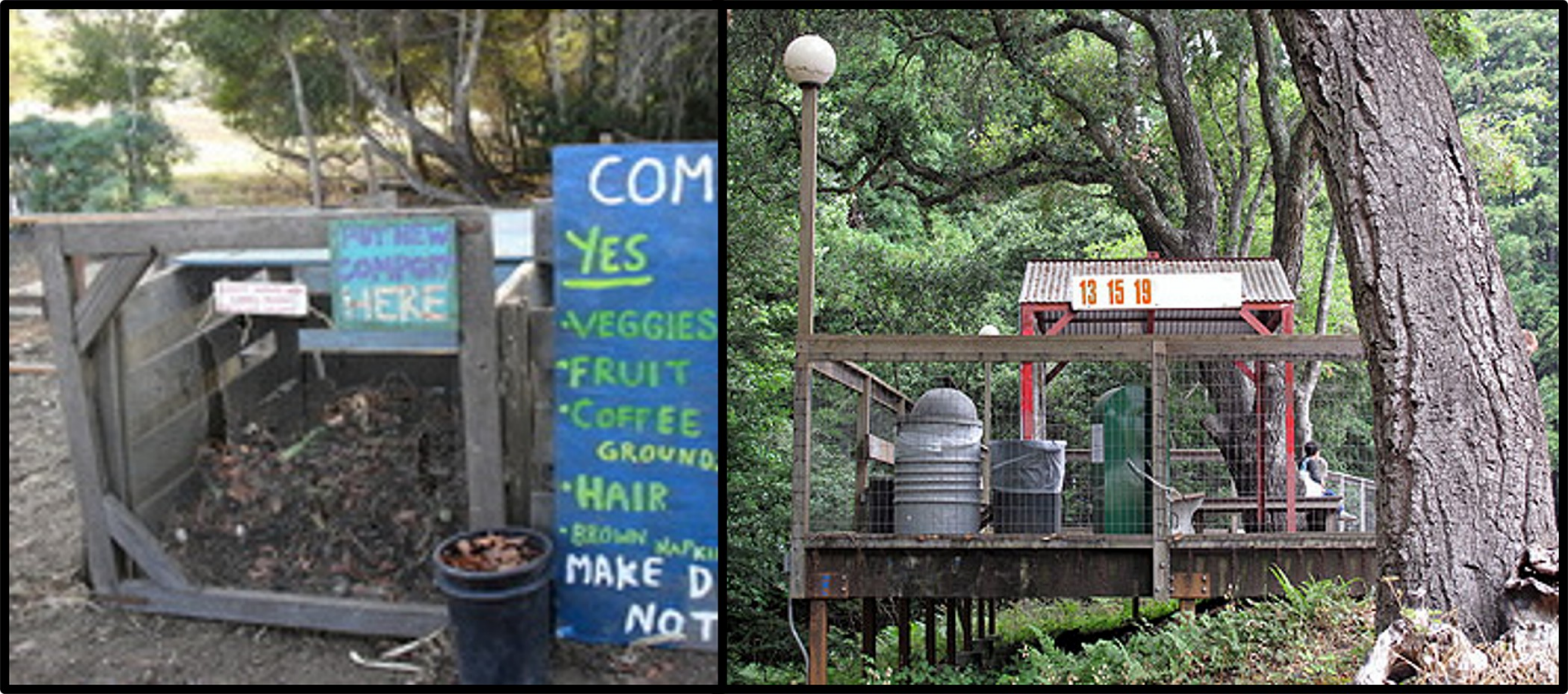
Examples of the trash vessels in the study. Depicted on the left is an open compost heap, and depicted on the right is an open trash can and a metal trash can with a swing lid.

In September of 2019, the UC Santa Cruz campus housed 9,339 students and had five active dining halls and eleven cafes [22]. Garbage was contained in plastic garbage bins on the back or side of all cafes and dining halls, save for one open compost heap in the west end (Fig 2). Standard metal trash bins were also placed around cafés and dining halls towards the front, all of which were either exposed or had a swing lid. The campus has recorded the presence of both invasive *Rattus rattus* and *Rattus norvegicus* presence in the surrounding area but lacks any specific distribution data [21]. During late March of 2020, Santa Cruz County issued a COVID-19 lockdown decree to slow the spread of Sars-CoV-2, which mandated that all non-essential work and public groupings be suspended [23]. On March 10, 2020, the UC Santa Cruz administration suspended in-person classes. The on-campus student population decreased to approximately 1,000 students, and only two dining halls remained open (without on-site dining) and limited their hours [22]. As a result, dumpsters and other sources of garbage were quickly emptied and not replaced, thus eliminating most sources of wildlife dietary supplementation. At pre- COVID lockdown levels, the campus generated 0.58 kgs/person/day of trash, of which 43% was organic matter (i.e., food scraps, compostable containers, soiled paper, etc.). Post- COVID lockdown, organic waste decreased by 72% [22]. While trace amounts of human supplementation still existed in post-COVID lockdown conditions, levels of refuse decreased to such a large extent that we maintain the experiment still functions as a removal experiment.

### Study design

We directly measured rat activity in pre- and post- COVID lockdown conditions via camera trapping. All fieldwork was conducted from November 23, 2019, to February 14, 2021, in two trapping periods across 15 sites and in all three habitats. Trapping in Period 1 (November 2019 –March 2020) served as a comparison for invasive rat activity under normal (pre-COVID lockdown) conditions and lasted for 18 weeks. Initial trapping objectives were to collect small mammal activity data (for both invasive and native species), around the university and the surrounding area. However, the shelter in place COVID-19 lockdown provided opportunistic circumstances for a food and waste removal experiment. Trapping during Period 2 (mid-October 2020 –February 2021) took place during the COVID 19 lockdown and was conducted at the same sites as Trapping Period 1. Trapping Period 2 lasted for 17 weeks and served as our experimental treatment approximately one year later during the COVID lockdown when human supplementation was significantly reduced.

To test our hypothesis that invasive rats are more dependent on human supplementation than native rodents, the first site in each trapping transect originated at a semi-exposed source of human refuse (dining hall dumpster, compost pile, etc.). We placed each sequential site in a direction away from the initial refuse source, towards natural habitat (Fig 1). To reduce the probability of capturing the same individual, each transect contained three sites spaced 75 m apart each, for a total transect length of 150 m (i.e., a site at 0 m, 75 m, and 150m, five transects, N = 15 total sites) [24]. To document small mammal activity, we used camera traps (Bushnell Trophy Cam; Bushnell Corp., Overland Park, KS, USA) that were left on-site for four consecutive nights, then collected and moved to the next transect (*N* = 12 trap nights per each of the five transects; 60 total trap nights per trapping period). We programmed the cameras to capture three images every 30 seconds once movement was detected. Each site was baited with 30 grams of sunflower seeds (*Helianthus annuus)* and rebaited every other night. All sites were originally open to the public (vehicles not allowed) but were closed from March 31, 2020 to September 2, 2020. We did not physically capture or collect any animals during the course of this project, and all data were collected non-invasively via camera trapping. We note that as we did not mark or capture any animals, we focus here on activity rather than abundance to avoid any resampling bias [25].

### Data analysis

**Fig 3:**
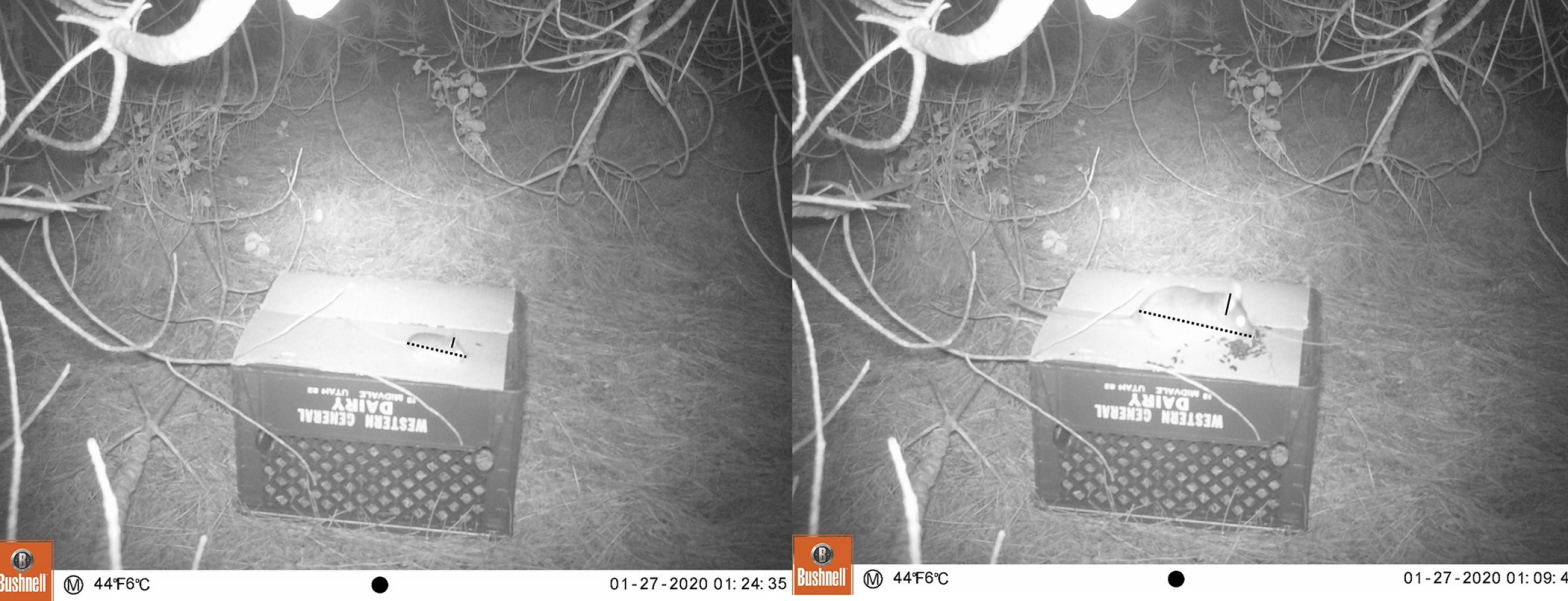
An example of rodent classification based on ear: body ratios (*Peromyscus* left, *Neotoma* right). Solid lines denote ear measurements, while dotted lines denote body measurements. All body measurements were taken from the tip of the snout to the base of the tail.

We tracked the activity of three genera of small mammal: deer mice *Peromyscus*, woodrats *Neotoma*, and the two species of invasive *Rattus*. *Peromyscus* and *Neotoma* served as our comparison group to the invasive rats as they were exposed to the same human refuse, but as they are both native genera, they might be more adapted to foraging on natural resources. We categorized all captured animals in Image J (Ver. 1.8.0). For each animal, we measured the profile ear and body length, then compared the ratio between the two. Both *Neotoma* and *Peromyscus* have a similar ear-to-body ratio, but the body size of *Peromyscus* is substantially smaller than *Neotoma* (Fig 3) [21, 26]. Conversely, *Rattus* ear-to-body ratio is far larger than both *Peromyscus* and *Neotoma* [27]. We exclusively used photos where the animal’s full body length and ear height were in view. After the animal was classified, we could then determine trapping success based on the rodent’s presence or absence in the frame.

To test the influence of habitat on invasive *Rattus* activity and to account for errors in camera trap detection, we created an occupancy model in the *unmarked* package in R [28]. We binary-coded invasive *Rattus* presence or absence with a 1 or a 0, respectively, and indexed habitat composition at each site as a covariate [28]. We included habitat as a covariate as we thought it would have potential to impact both occupancy and detection by providing more resources or places to hide, respectively. We treated each individual night as an independent survey trial, which yielded four repeated surveys at each site. Finally, we back-transformed our detection and occupancy estimates and fitted 95% confidence intervals. To test the influence of habitat on invasive rat activity, we created three models: one with variable occupancy, one with variable detection, and one with both variable occupancy and variable detection as a function of habitat.

We created a number of mixed linear regression models beforehand to examine the possible impacts of refuse presence, habitat type, distance to refuse, and native small mammal presence on rat activity [29]. We also included trap night (N = 120) and trap site (n = 15) as random effects. We predicted that rat activity would decrease in the absence of human refuse regardless of habitat type or native small mammal presence. To measure the effects of human supplementation on native small mammals, we also created a second set of mixed linear regression models with the same predictors to serve as our control. Conversely, here we hypothesized that extra supplementation would have no effect on small mammal activity.

We included habitat as a predictor based on its potential to provide excess food or shelter from predators [30]. While certain habitats (e.g. mixed oak), might provide more access to natural resources, we expected invasive rats to rely so heavily on human refuse that this extra supplementation would not offset decreases in rat activity. Similarly, we included the presence or absence of native small mammals as a predictor to incorporate resource competition between the two species [31]. However, while we expected small mammals would provide some amount of competition, we predicted the influence of native small mammals to be negligent as invasive rats would be used to relying on human refuse instead of natural resources. Finally, we included the distance to human refuse as a predictor, as we expected rat activity to decrease further away from human supplementation. We tested for collinearity between our predictors with a variance inflation factor test (VIF) and found an absence of collinearity. We then created a model candidate set that included the effects of these variables on invasive rat activity or native small mammal activity and evaluated the efficacy of the candidate models with AIC values [32]. We selected our top performing models based on the lowest AIC value [33].

## Results

### Small mammal distribution pre and post-COVID lockdown

**Fig 4:**
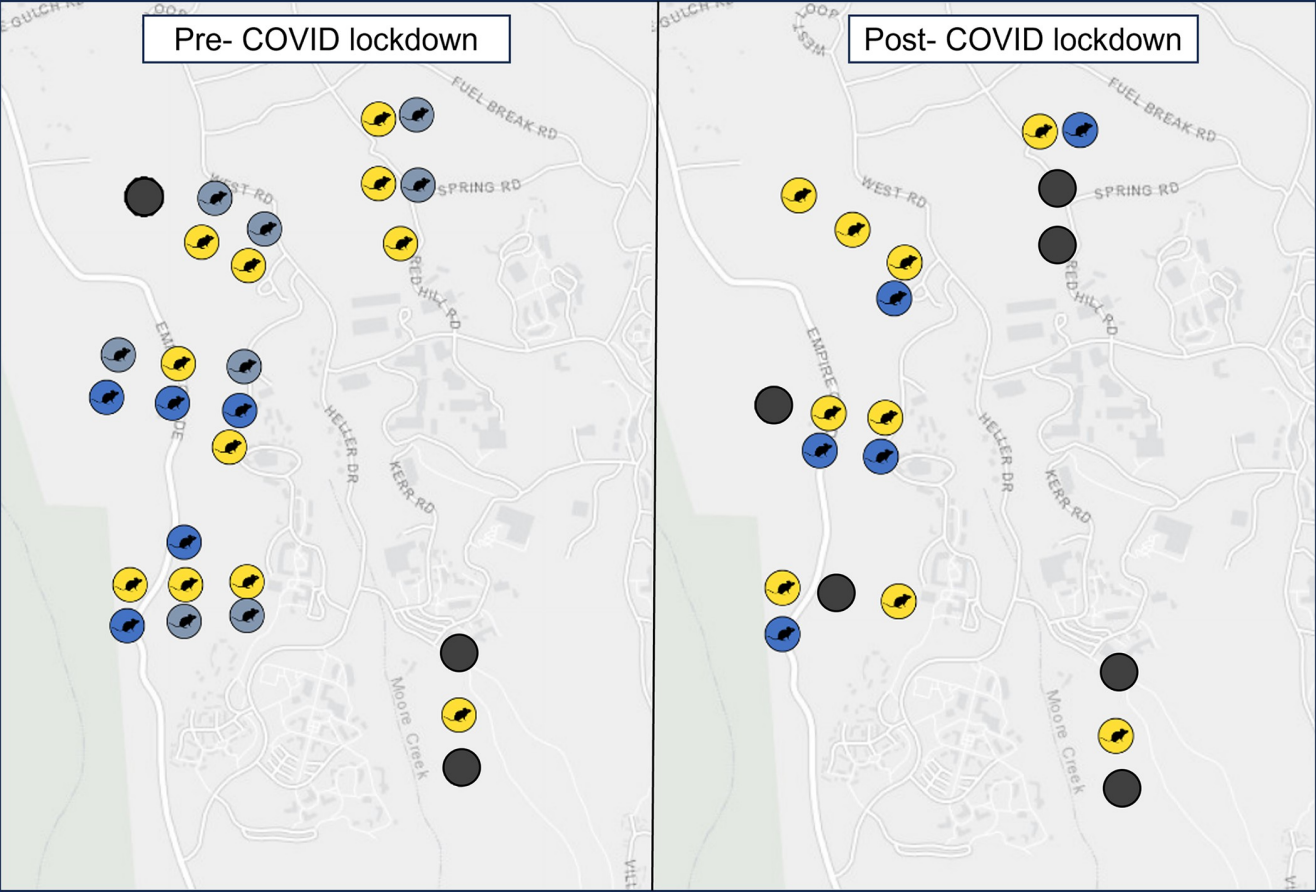
Distribution of rodent presence or absence in pre- and post- COVID lockdown conditions. Light gray circles indicate the presence of invasive *Rattus*, yellow circles indicate the presence of *Peromyscus*, blue circles indicate the presence of *Neotoma*, and dark gray circles indicate the absence of any rodent detections.

**Fig 5:**
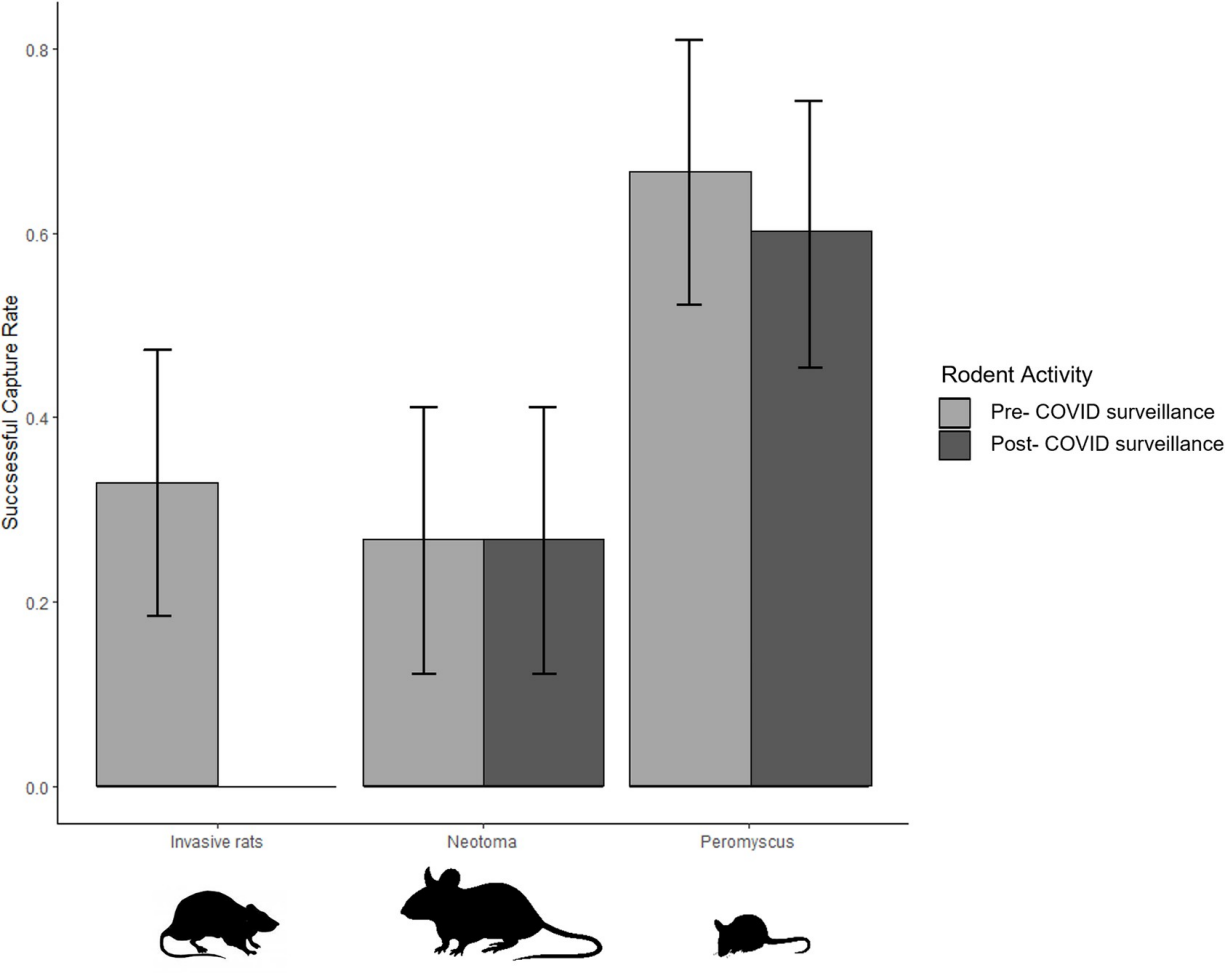
Total site occupancy capture rates in *Neotoma*, *Peromyscus*, and invasive rats between trapping Periods 1 and 2. “Successful capture rate” indicates the proportion of sites where the animal was captured. Invasive rat activity decreased between periods (ρ = 0.33 successful capture rate during Trapping Period 1, ρ = 0 successful capture rate during Trapping Period 2).

After 14 weeks of trapping, we detected small mammal species at 80% of our sites in the pre-COVID lockdown period. We detected invasive *Rattus* species at five sites (33.3% success rate), and we detected *Peromyscus* at 10 sites (66.6% success rate). Finally, we detected *Neotoma* at four sites (26.6% success rate). At each site where we detected invasive *Rattus*, we also detected other small mammal species: 40% of invasive *Rattus* occupied sites were shared with *Neotoma* and 60% of invasive *Rattus*-occupied sites were shared with *Peromyscus* (Fig 4). After the decrease of human refuse in post- COVID lockdown conditions, we no longer detected invasive *Rattus* at any of our sites. Overall, we detected native small mammal activity at 80% of our sites in the post-COVID lockdown period. We detected *Peromyscus* at 60% of our sites and *Neotoma* at 26.66% of our sites (Fig 5).

### Habitat preference and rodent distribution

As there were zero detections to test for invasive rat habitat activity in post-COVID lockdown conditions, we used exclusively pre-COVID lockdown data for our habitat-covariate occupancy model. We found that habitat composition did not influence invasive rat activity, as invasive *Rattus* activity was relatively uniformly distributed throughout all habitat types (Tables 1 and 2).

**Table 1:** Estimated proportion of total sites occupied by invasive *Rattus*, followed by the detection probability. All results are back transformed linear combinations and fitted with a 95% confidence interval.

	Estimate	SE	LinComb	CI low	CI high
Occupancy	0.363	0.131	-0.561	-1.666	0.54480
Detection	0.639	0.144	0.571	-0.3965	1.5367

**Table 2:** Occupancy model results using habitat as a covariate that affects occupancy, detection probability, or both (pre-COVID conditions only). We tested three models: constant detection and variable occupancy, variable detection and constant occupancy, and variable detection and variable occupancy as a function of habitat.

	Estimate	SE	Z	p-value	AIC
Variable Occupancy	-0.954	0.896	-1.065	0.287	47.835
Variable detection	0.787	1.03	0.763	0.446	49.423
Variable Occupancy + Detection	0.847	1.05	0.807	0.420	49.159

Our top performing invasive rat model included both trapping period and habitat as predictors, and our top performing native small mammal model included trapping period, habitat, and distance to human refuse (Tables 3 and 4). When we compared invasive rat activity in pre- and post-COVID lockdown conditions, we found the decrease in *Rattus* activity to be highly significant (p < 0.01). In contrast, we did not find any significant changes in activity in our native small mammal model in pre- and post- COVID lockdown conditions (p = 0.6).

**Table 3:** Top three performing mixed linear regression models for the influence of human supplementation on invasive rat activity, including AIC values and the difference between each model and the lowest AIC (ΔAIC). All models included trap night and site as random effects.

Fixed effects	AIC	ΔAIC
Human supplementation + habitat	49.44	0
Human supplementation + small mammal activity	49.51	0.06
Human supplementation + native small mammal activity + habitat	50.43	0.99

**Table 4:** Top three performing mixed linear regression models for the influence of human supplementation on native small mammal activity, including AIC values and the difference between each model and the lowest AIC (ΔAIC). All models included trap night and site as random effects.

Fixed effects	AIC	ΔAIC
Habitat + distance to human supplementation + Human supplementation	-107.6	0
Distance to human supplementation + Human supplementation	-101.9	5.7
Habitat + Human supplementation	-101.8	5.8

## Discussion

In this experiment, we found that the activity of peri-urban invasive species of *Rattus* is highly correlated with available human refuse. We observed little to no change in native foragers’ activity (*Peromyscus* and *Neotoma*), with a slight decrease in *Peromyscus* and no change in *Neotoma* activity (Fig 5), indicating the removal of human supplementation had no significant effect on native rodent populations. This decrease in invasive rat activity suggests that the rats used this human supplementation to sustain their population levels. We observed this pattern across all habitat types, indicating that human-obligate foraging is not influenced by habitat composition (Table 2). Our results are bolstered by previous work, which found that urban populations of rats are also known to congregate around garbage receptacles. While urban populations of invasive rats have a comparatively faster growth rate and earlier maturation compared to purely wild populations of invasive rats, urban and peri-urban populations lack the ability to quickly recover from a disturbance [15–17, 34, 35]. Peri-urban rat populations are extremely sensitive to sudden resource loss, and these changes can alter the population equilibrium [15]. This is coupled with an extremely high mortality rate due to a variety of factors (e.g., competition, resource limitation, etc.) [9, 15]. Similarly, peri-urban and urban rats display high rates of neophobia, or resistance to new stimuli, in response to novel food resources. These periods of neophobia can be overcome, but are usually done so in a gradual manner, wherein the rat takes increasing amounts of food each trip [36].

Humans have a long history of conflict with invasive rats, and several measures have been taken against invasive rat populations [37–39]. Our study suggests that strict food waste management or removal could have similar and more targeted impacts as other methods of rodent control, including rodenticides. Urban areas have often employed rodenticides as a means of rat control [40, 41]. While the use of these chemicals can have a short-term reduction in rodents it is likely not an effective control method on without infrastructure modifications [42, 43]. Invasive rat populations are resilient to poisoning events, as their reproductive rates and neophobic behavior can quickly adjust after such events [34, 36]. Additionally, rodenticides have many indirect effects, as mammals that scavenge on poisoned rat carcasses are at a higher risk of secondary poisoning [35, 44, 45]. Our results provide experimental support that communities that include strict food waste management or removal as part of their rat control programs may see declines in invasive rat populations [34, 41].

We note that we did not track invasive rat activity outside our study area. While human supplementation decreases could have led to large population reductions, the opportunistic foraging behavior of rats could suggest that the rats moved to areas with a higher patch quality or more abundant temporary resources [46]. While camera traps have high accuracy in estimating population levels, other ephemeral seasonal variations (e.g. fluctuations in predator populations, change in climate) that we did not capture could have confounded the results [47]. Similarly, we solely recorded invasive and native rodent activity via camera traps, and did not live-capture any rodents or compare with any local pest control data, which could potentially limit the scope of this study [17]. Despite these limitations, our results indicate that peri-urban invasive rat populations may rely on human refuse to maintain a stable population, and proper control and management of human refuse can eradicate a local population regardless of its extension into natural habitat.

## Conclusion

In our non-invasive removal experiment, we directly measured rat activity before and after COVID lockdown conditions and found evidence that peri-urban invasive rat population levels are highly correlated with human refuse quantities. Furthermore, our results imply that invasive rats fail to maintain their former population levels purely on natural resources, regardless of patch quality. These results offer conservation implications, as they indicate that proper food waste management is likely to be more effective and more targeted at controlling invasive rats.
